# Immune Checkpoint Inhibitor Pneumonitis Complicated by Invasive Pulmonary Aspergillosis in COPD: Diagnostic and Therapeutic Challenges

**DOI:** 10.1002/ccr3.72755

**Published:** 2026-05-24

**Authors:** Chukwuka Elendu, Ayi T. Debua, Emeka H. Okolo, Halimat O. Sadiq

**Affiliations:** ^1^ Federal University Teaching Hospital Owerri Nigeria; ^2^ University of Calabar Teaching Hospital Calabar Nigeria; ^3^ The Park Hospital Nottingham UK; ^4^ University of Lagos Lagos Nigeria

**Keywords:** checkpoint inhibitor‐associated pneumonitis, chronic obstructive pulmonary disease, immune checkpoint inhibitors, invasive pulmonary aspergillosis, metagenomic next‐generation sequencing

## Abstract

Checkpoint inhibitor‐associated pneumonitis complicated by invasive pulmonary aspergillosis represents a diagnostic challenge in ICI‐treated patients, particularly those with COPD receiving corticosteroid therapy. Persistent or worsening respiratory abnormalities despite immunosuppressive treatment should prompt reassessment for superimposed fungal infection, including bronchoscopy, BALF analysis, and microbiologic testing to facilitate diagnosis and targeted therapy.

Dear Editor,

We read with great interest the recently published case report titled “A Case Report of Immune Checkpoint Inhibitor‐Associated Pneumonia Complicated by Invasive Pulmonary Aspergillosis in a Patient With Chronic Obstructive Pulmonary Disease” [1], which highlights the diagnostic complexities arising from the coexistence of immune‐related pulmonary toxicity and fungal superinfection during immunotherapy.

The expanding use of immune checkpoint inhibitors (ICIs) has improved survival outcomes across several advanced malignancies, including lung cancer, melanoma, and gastrointestinal tumors. Agents targeting programmed cell death protein 1 (PD‐1), programmed death ligand 1 (PD‐L1), and cytotoxic T‐lymphocyte‐associated protein 4 (CTLA‐4) restore T‐cell‐mediated immune surveillance to exert antitumor effects [1, 2]. However, ICIs can produce immune‐related adverse events (irAEs) affecting multiple organ systems, with pulmonary toxicity, particularly checkpoint inhibitor‐associated pneumonitis (CIP), representing one of the most serious complications because of its links to respiratory failure, treatment interruption, and mortality [1, 2]. Although the reported incidence of CIP is approximately 5%–10%, the risk may be greater in patients with pre‐existing lung disease or those receiving combination immunotherapy [1, 2].

The patient described in the original report [1] possessed several risk factors for pulmonary complications, including advanced age, chronic obstructive pulmonary disease (COPD), prior chemotherapy exposure, and prolonged immunomodulatory therapy. COPD is increasingly recognized as a risk factor for both CIP and secondary fungal infection, as structural lung abnormalities, impaired mucociliary clearance, chronic airway inflammation, recurrent bacterial colonization, and altered innate immunity increase susceptibility to fungal colonization and invasive disease [3, 4]. In addition, emphysema and pulmonary bullae may obscure early inflammatory changes on imaging, making differentiation between immune‐mediated pneumonitis and fungal superinfection especially challenging, particularly when corticosteroid therapy transiently improves symptoms.

An important strength of the reported case lies in its demonstration of the evolving nature of lung pathology during immunotherapy. The patient's initial imaging findings and symptomatic improvement following corticosteroid therapy supported a diagnosis of CIP; however, subsequent clinical deterioration, worsening infiltrates, and respiratory failure suggested a superimposed process. This underscores the need to reassess patients with presumed CIP who deteriorate despite treatment, particularly for infectious complications, pulmonary embolism, resistant inflammation, or tumor progression (Figure 1).

**FIGURE 1:**
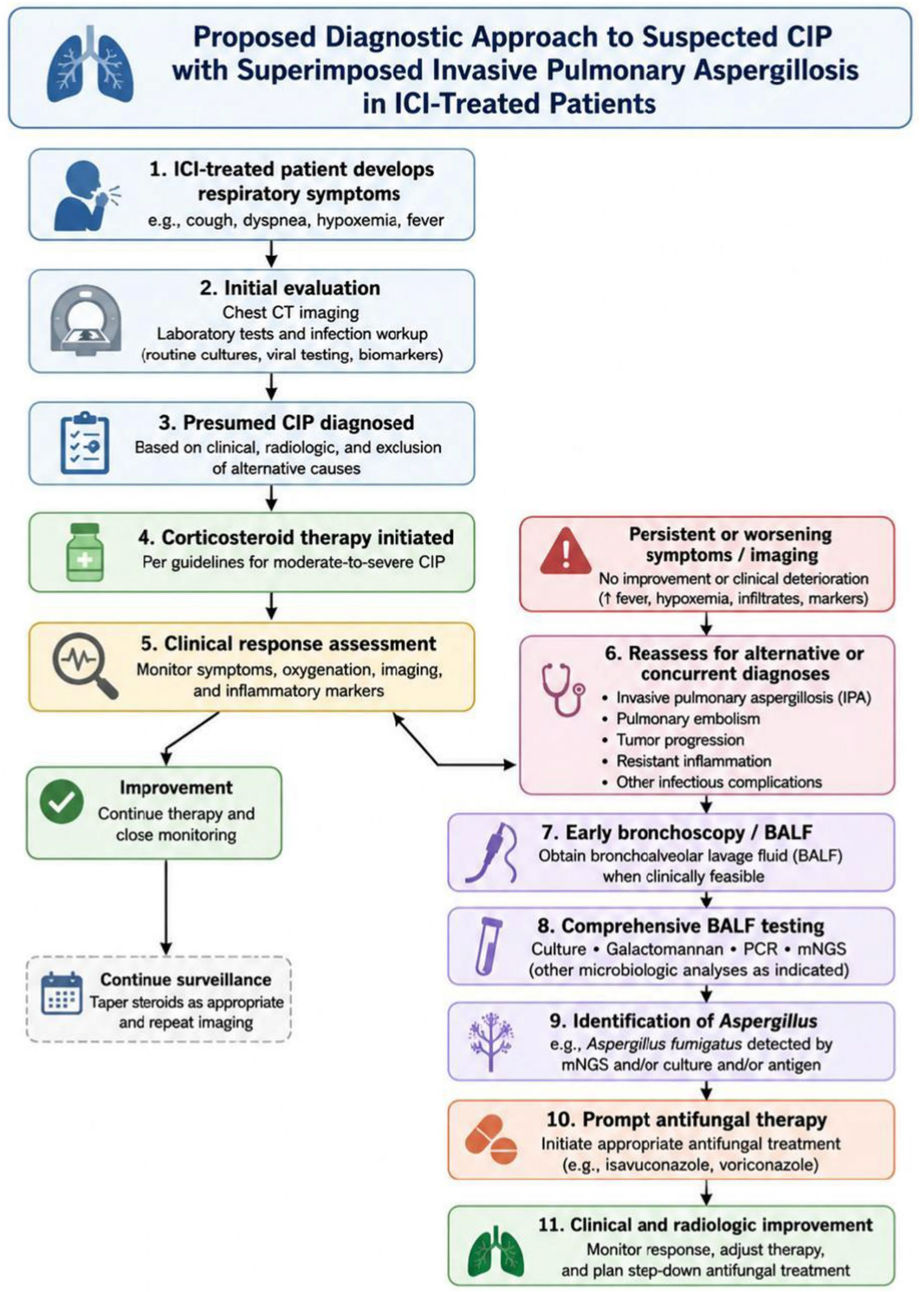
Proposed diagnostic approach for suspected CIP with superimposed IPA in ICI‐treated patients.

The diagnosis is further complicated by the substantial radiologic convergence between CIP and IPA (Figure 2). CIP commonly presents with bilateral ground‐glass opacities, organizing pneumonia patterns, interstitial infiltrates, diffuse alveolar damage, or hypersensitivity‐like changes [1, 5], whereas IPA may manifest as nonspecific consolidations, nodules, bronchopneumonia, cavitary lesions, or diffuse infiltrative changes [6, 7]. Moreover, classical signs such as the halo sign or air‐crescent sign are often absent in non‐neutropenic patients, including those with COPD [6, 8]. Consequently, reliance on imaging alone may delay recognition of concomitant IPA, particularly when radiologic abnormalities persist or progress after corticosteroid initiation.

**FIGURE 2:**
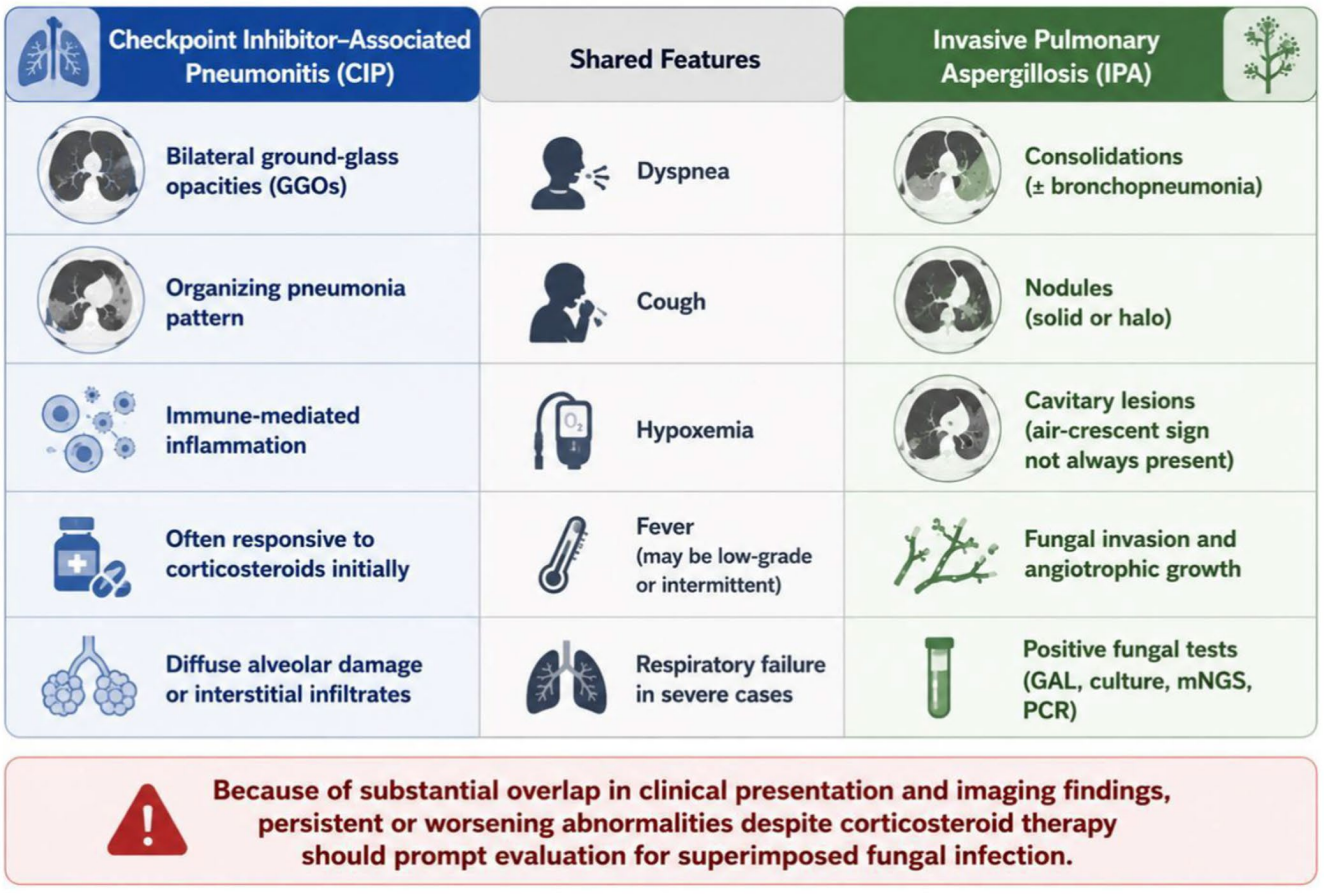
Overlapping clinical and radiologic features of CIP and IPA.

In patients with severe respiratory compromise, bronchoalveolar lavage fluid (BALF) examination may provide a safer alternative to invasive lung biopsy when integrated with microbiological culture, galactomannan testing, polymerase chain reaction assays, and metagenomic next‐generation sequencing (mNGS). Here, mNGS identified *Aspergillus fumigatus* with a high sequence count and relative abundance, findings subsequently confirmed by culture positivity. This supports growing evidence that mNGS may facilitate earlier recognition of invasive fungal infections in patients without neutropenia, in whom conventional biomarkers often have limited sensitivity [9, 10].

The growing recognition of IPA in non‐neutropenic patients also warrants attention. Although traditionally associated with severe immunocompromise, including hematologic malignancies, profound neutropenia, and organ transplantation [11, 12], IPA is now frequently reported in critically ill patients without classical immunosuppression, particularly those with COPD, viral pneumonia, prolonged steroid exposure, or intensive care unit admission [3, 4, 13]. COPD patients receiving systemic corticosteroids appear especially vulnerable because these agents impair macrophage activation, neutrophil function, and fungal clearance while promoting fungal proliferation [4]. In this patient, corticosteroid therapy for presumed CIP likely facilitated accelerated *Aspergillus* invasion, highlighting the therapeutic challenge that treatment essential for CIP management may simultaneously increase susceptibility to secondary fungal infection.

The transient symptomatic improvement following corticosteroid initiation is clinically instructive, as temporary stabilization may falsely reassure clinicians and delay recognition of invasive fungal disease. Although inflammatory suppression can reduce pulmonary edema and immune‐mediated tissue injury, fungal proliferation may continue unchecked. The patient's subsequent deterioration likely reflected progression of occult *Aspergillus* infection exacerbated by steroid‐induced immunosuppression. Clinicians managing CIP should therefore maintain a high index of suspicion for fungal superinfection in patients with incomplete radiologic resolution, persistent fever, worsening hypoxemia, rising inflammatory markers, or new infiltrates despite immunosuppressive therapy.

The role of cadonilimab in this case is also noteworthy. As a PD‐1/CTLA‐4 bispecific antibody, cadonilimab represents a newer generation of immunotherapeutic agents designed to enhance antitumor activity while potentially reducing systemic toxicity [14]. However, enhanced immune activation may increase the risk of irAEs, including pneumonitis, and combination checkpoint inhibition has been associated with higher rates of lung toxicity than monotherapy [1, 2]. As these agents become more widely used, clinicians may increasingly encounter complex adverse events involving concurrent inflammatory and infectious processes, underscoring the need for multidisciplinary collaboration among oncologists, pulmonologists, radiologists, infectious disease specialists, and intensivists.

Another important aspect of this report involves the limitations of conventional fungal biomarkers in non‐neutropenic patients. The initially negative serum galactomannan result reflects a recognized challenge in IPA diagnosis among patients with COPD, in whom these assays often demonstrate lower sensitivity because angioinvasion may be less pronounced than in neutropenic hosts [8]. Consequently, negative biomarkers should not exclude IPA when clinical suspicion remains high, and BALF galactomannan testing, fungal culture, and mNGS may provide greater diagnostic yield in such settings [10, 11, 12, 13, 14, 15]. These findings support early lower respiratory tract sampling in suspected CIP complicated by invasive fungal infection whenever clinically feasible.

Progression to respiratory failure further highlights the life‐threatening potential of combined CIP and IPA. Respiratory deterioration during immunotherapy is often multifactorial, reflecting concurrent infection, immune‐mediated injury, impaired gas exchange, and underlying structural lung disease. COPD patients possess limited pulmonary reserve and may rapidly decompensate when exposed to diffuse inflammatory or infectious insults. In this case, high‐flow oxygen therapy and prompt antifungal treatment resulted in marked clinical and radiologic improvement, underscoring the value of timely reevaluation and targeted antimicrobial therapy.

Although triazole antifungals remain first‐line therapy for IPA, with voriconazole historically regarded as the preferred agent [12], isavuconazole has emerged as an attractive alternative because of its favorable safety profile, including reduced hepatotoxicity, lower risk of QT prolongation, and fewer drug–drug interactions [12]. These advantages may be especially relevant in oncology patients receiving multiple medications. The subsequent transition to oral itraconazole after clinical stabilization further highlights the value of individualized step‐down antifungal therapy based on treatment response and tolerability.

Beyond the individual clinical details, this report raises important questions regarding surveillance for fungal superinfection during treatment of severe irAEs. Current guidelines emphasize prompt corticosteroid initiation for moderate‐to‐severe CIP [5, 16], yet recommendations for fungal surveillance in high‐risk patients remain limited. COPD patients receiving prolonged corticosteroid therapy after ICI exposure may represent a particularly vulnerable subgroup requiring closer microbiologic monitoring. Baseline fungal risk assessment, early bronchoscopy in deteriorating patients, serial imaging, and fungal biomarkers may improve early detection of IPA in selected cases. Routine antifungal prophylaxis cannot currently be recommended because of insufficient evidence, although future studies may clarify whether certain high‐risk populations could benefit from preventive strategies.

Although ICIs restore T‐cell activity against tumor cells, they may also dysregulate alveolar immune homeostasis. The inflammatory milieu associated with CIP can damage epithelial barriers, impair alveolar macrophage function, and increase susceptibility to infection while fungal invasion may further amplify inflammation and complicate interpretation of treatment‐related toxicity. This bidirectional interaction between immune dysregulation and fungal infection likely contributed to the patient's complex presentation. Improved understanding of these mechanisms may facilitate development of biomarkers capable of distinguishing inflammatory pneumonitis from superimposed infection.

Repeated clinical reassessment remains essential and should take precedence over reliance on an initial diagnostic impression. In contemporary oncologic practice, new pulmonary infiltrates in ICI‐treated patients may be prematurely attributed to immune‐related toxicity, although respiratory complications in cancer patients are often multifactorial. Infection, aspiration, drug toxicity, pulmonary edema, radiation injury, tumor progression, and thromboembolic disease may coexist, emphasizing the need to avoid diagnostic anchoring, particularly when patients fail to improve as expected.

Although the limitations acknowledged by the authors, including the absence of early bronchoscopy, incomplete fungal biomarker evaluation, and lack of histopathologic confirmation, limit definitive assessment of the temporal relationship between CIP and IPA, the overall clinical presentation, microbiologic findings, radiologic progression, and response to antifungal therapy strongly support the diagnosis of IPA complicating CIP. In real‐world practice, many critically ill patients cannot safely undergo invasive procedures, necessitating integration of clinical, radiologic, and microbiologic data. Conventional culture‐based diagnostics may require prolonged turnaround times and fail to detect fastidious organisms, whereas mNGS enables comprehensive pathogen identification directly from clinical specimens and may be particularly valuable in immunocompromised or diagnostically complex patients [9, 10]. Despite challenges related to cost, standardization, availability, and differentiation between colonization and infection, mNGS may significantly enhance future diagnostic pathways for severe complications during immunotherapy.

Intensive care physicians increasingly encounter severe irAEs requiring ventilatory support, immunosuppression, and multidisciplinary care. Concurrent inflammatory pneumonitis and fungal superinfection further complicate treatment decisions, as clinicians must balance immunosuppressive therapy with infection control. Delayed recognition of invasive fungal disease may lead to fulminant respiratory failure and increased mortality, underscoring the need for early infectious disease consultation and consideration of invasive diagnostic sampling in deteriorating patients.

As cancer survival improves with immunotherapy, delayed and atypical treatment‐related complications will likely become more common across multiple specialties. Education regarding irAEs and associated fungal infections should become an integral component of oncologic, pulmonary, and critical care training. In addition, prospective registries and multicenter studies are needed to better define the incidence, risk factors, outcomes, and optimal treatment strategies for concomitant CIP and IPA.

In conclusion, immune checkpoint inhibitor pneumonitis complicated by invasive pulmonary aspergillosis represents an important diagnostic challenge, particularly in patients receiving corticosteroid therapy. Clinicians should maintain a high index of suspicion for superimposed invasive fungal infection in patients with worsening respiratory status during treatment for presumed CIP. As ICI use continues to expand globally, heightened awareness of these concurrent complications will be essential for improving patient outcomes.

## Author Contributions

**Chukwuka Elendu:** conceptualization, investigation, data curation, methodology, formal analysis, writing – review and editing, writing – original draft, visualization, validation, project administration, supervision. **Ayi T. Debua:** investigation, validation, writing – review and editing. **Emeka H. Okolo:** formal analysis, writing – review and editing, validation. **Halimat O. Sadiq:** validation, visualization, writing – review and editing.

## Funding

The authors have nothing to report.

## Ethics Statement

Ethical approval was not required, as this letter does not involve new patient data, human subject research, or original clinical investigation.

## Consent

Written informed consent for publication was obtained where applicable. This letter does not include new patient data.

## Conflicts of Interest

The authors declare no conflicts of interest.

## Data Availability

Data sharing not applicable to this article as no datasets were generated or analysed during the current study.

## References

[1] S. Lin , “A Case Report of Immune Checkpoint Inhibitor‐Associated Pneumonia Complicated by Invasive Pulmonary Aspergillosis in a Patient With Chronic Obstructive Pulmonary Disease,” Clinical Case Reports 14, no. 4 (2026): e72470, 10.1002/ccr3.72470. 41953097 PMC13053302

[2] W. T. Atchley , C. Alvarez , S. Saxena‐Beem , et al., “Immune Checkpoint Inhibitor‐Related Pneumonitis in Lung Cancer: Real‐World Incidence, Risk Factors, and Management Practices Across Six Health Care Centers in North Carolina,” Chest 160, no. 2 (2021): 731–742, 10.1016/j.chest.2021.02.032. 33621599 PMC8411447

[3] S. Molinos‐Castro , P. M. Pesqueira‐Fontán , S. Rodríguez‐Fernández , et al., “Clinical Factors Associated With Pulmonary Aspergillosis in Patients With Chronic Obstructive Pulmonary Disease,” Enfermedades Infecciosas y Microbiología Clínica (English ed.) 38, no. 1 (2020): 4–10, 10.1016/j.eimc.2019.06.007. 31405617

[4] P. Bulpa , A. Dive , and Y. Sibille , “Invasive Pulmonary Aspergillosis in Patients With Chronic Obstructive Pulmonary Disease,” European Respiratory Journal 30, no. 4 (2007): 782–800, 10.1183/09031936.00062206. 17906086

[5] J. R. Brahmer , H. Abu‐Sbeih , P. A. Ascierto , et al., “Society for Immunotherapy of Cancer (SITC) Clinical Practice Guideline on Immune Checkpoint Inhibitor‐Related Adverse Events,” Journal for Immunotherapy of Cancer 9, no. 6 (2021): e002435, 10.1136/jitc-2021-002435. 34172516 PMC8237720

[6] J. Heylen , Y. Vanbiervliet , J. Maertens , B. Rijnders , and J. Wauters , “Acute Invasive Pulmonary Aspergillosis: Clinical Presentation and Treatment,” Seminars in Respiratory and Critical Care Medicine 45, no. 1 (2024): 69–87, 10.1055/s-0043-1777769. 38211628

[7] C. Lim , J. B. Seo , S. Y. Park , et al., “Analysis of Initial and Follow‐Up CT Findings in Patients With Invasive Pulmonary Aspergillosis After Solid Organ Transplantation,” Clinical Radiology 67, no. 12 (2012): 1179–1186, 10.1016/j.crad.2012.02.018. 22766482

[8] L. Liu , Y. Gu , Y. Wang , K. Shen , and X. Su , “The Clinical Characteristics of Patients With Nonneutropenic Invasive Pulmonary Aspergillosis,” Frontiers in Medicine (Lausanne) 8 (2021): 631461, 10.3389/fmed.2021.631461. PMC7917130 33659265

[9] Y. Li , B. Sun , X. Tang , et al., “Application of Metagenomic Next‐Generation Sequencing for Bronchoalveolar Lavage Diagnostics in Critically Ill Patients,” European Journal of Clinical Microbiology & Infectious Diseases 39, no. 2 (2020): 369–374, 10.1007/s10096-019-03734-5. 31813078 PMC7102353

[10] N. Zhu , D. Zhou , W. Xiong , X. Zhang , and S. Li , “Performance of mNGS in Bronchoalveolar Lavage Fluid for the Diagnosis of Invasive Pulmonary Aspergillosis in Non‐Neutropenic Patients,” Frontiers in Cellular and Infection Microbiology 13 (2023): 1271853, 10.3389/fcimb.2023.1271853. 38029249 PMC10644336

[11] J. P. Donnelly , S. C. Chen , C. A. Kauffman , et al., “Revision and Update of the Consensus Definitions of Invasive Fungal Disease From the European Organization for Research and Treatment of Cancer and the Mycoses Study Group Education and Research Consortium,” Clinical Infectious Diseases 71, no. 6 (2020): 1367–1376, 10.1093/cid/ciz1008. 31802125 PMC7486838

[12] T. F. Patterson , G. R. Thompson, 3rd , D. W. Denning , et al., “Practice Guidelines for the Diagnosis and Management of Aspergillosis: 2016 Update by the Infectious Diseases Society of America,” Clinical Infectious Diseases 63, no. 4 (2016): e1–e60, 10.1093/cid/ciw326. 27365388 PMC4967602

[13] T. Mir , M. Uddin , A. Khalil , et al., “Mortality Outcomes Associated With Invasive Aspergillosis Among Acute Exacerbation of Chronic Obstructive Pulmonary Disease Patient Population,” Respiratory Medicine 191 (2022): 106720, 10.1016/j.rmed.2021.106720. 34959147

[14] M. Z. Dong , M. Cui , E. B. Zhu , et al., “Recent Progress in Cadonilimab Research for Oncology Applications,” Frontiers in Immunology 16 (2026): 1694490, 10.3389/fimmu.2025.1694490. 41613151 PMC12847384

[15] C. Lass‐Flörl , “How to Make a Fast Diagnosis in Invasive Aspergillosis,” Medical Mycology 57, no. Suppl 2 (2019): S155–S160, 10.1093/mmy/myy103. 30816965

[16] J. Haanen , M. Obeid , L. Spain , et al., “Management of Toxicities From Immunotherapy: ESMO Clinical Practice Guideline for Diagnosis, Treatment and Follow‐Up,” Annals of Oncology 33, no. 12 (2022): 1217–1238, 10.1016/j.annonc.2022.10.001. 36270461

